# Patient characteristics and outcomes associated with adherence to the low PEEP/FIO2 table for acute respiratory distress syndrome

**DOI:** 10.1038/s41598-021-94081-z

**Published:** 2021-07-16

**Authors:** Kay Choong See, Juliet Sahagun, Juvel Taculod

**Affiliations:** 1 Division of Respiratory & Critical Care Medicine, Department of Medicine, National University Hospital, 1E Kent Ridge Road, NUHS Tower Block Level 10, Singapore, 119228 Singapore; 2 Department of Medicine, Yong Loo Lin School of Medicine, National University of Singapore, Singapore, Singapore; 3 Division of Critical Care—Respiratory Therapy, National University Hospital, Singapore, Singapore

**Keywords:** Respiratory tract diseases, Therapeutics

## Abstract

It remains uncertain how best to set positive end-expiratory pressure (PEEP) for mechanically ventilated patients with the acute respiratory distress syndrome (ARDS). Among patients on low tidal volume ventilation (LTVV), we investigated if further adherence to the low PEEP/FIO2 (inspired oxygen fraction) table would be associated with better survival compared to nonadherence. Patients with ARDS, admitted directly from the Emergency Department to our 20-bed Medical Intensive Care Unit (ICU) from August 2016 to July 2017, were retrospectively studied. To determine adherence to the low PEEP/FIO2 table, PEEP and FIO2 12 h after ICU admission were used, to reflect ventilator adjustments by ICU clinicians after initial stabilization. Logistic regression was used to analyze hospital mortality as an outcome with adherence to the low PEEP/FIO2 as the key independent variable, adjusted for age, APACHE II score, initial P/F ratio and initial systolic blood pressure. 138 patients with ARDS were analysed. Overall adherence to the low PEEP/FIO2 table was 75.4%. Among patients on LTVV, nonadherence to the low PEEP/FIO2 table was associated with increased mortality compared to adherence (adjusted odds ratio 4.10, 95% confidence interval 1.68–9.99, P = 0.002). Patient characteristics at baseline were not associated with adherence to the low PEEP/FIO2 table.

## Introduction

To improve survival of patients with acute respiratory distress syndrome (ARDS)^1^, an optimal mechanical ventilation strategy includes low tidal volume ventilation (LTVV)^2^ and avoidance of either hypoxemia or hyperoxemia^3^. However, it remains uncertain how best to set positive end-expiratory pressure (PEEP)^4–6^. If the PEEP is set too low, the patient runs the risk of ventilator-induced lung injury from cyclic opening and closing of alveoli. If the PEEP is set too high, the patient runs the risk of alveolar over-distension^7^ (possibly leading to barotrauma and biotrauma), and excessive intrathoracic pressure^8^ (possibly leading to reduced venous return, increased right ventricular afterload and systemic hypotension).

Several methods to set PEEP are available. One way is to target an end-expiratory transpulmonary pressure of 0–10 cm H2O to reduce cyclic alveolar collapse, and an end-inspiratory transpulmonary pressure ≤ 25 cm H2O to reduce alveolar overdistension^9^. This requires measurement of pleural pressure using an esophageal balloon catheter. Other ways to set PEEP are by directly visualizing improvement of lung aeration via electrical impedance tomography^10^ or lung ultrasound^11^, by using pressure–volume curves to set PEEP above the lower inflection point^12^, by maximizing static respiratory system compliance^13^, or by assessing the recruitment-to-inflation ratio^14^. However, randomized controlled trials have not supported the transpulmonary pressure approach^15^ and are inconclusive for the other approaches^16^.

PEEP levels can also be recommended based on the inspired oxygen fraction (FIO2), with a higher FIO2 requirement calling for higher PEEP. PEEP/FIO2 tables have been created to guide clinicians^17^, with two variants available (Table 1)^4,18^. For the same FIO2, the low PEEP/FIO2 table would recommend lower PEEP settings compared to the high PEEP/FIO2 table. Nonetheless, randomized trials of PEEP titration using the low versus high PEEP/FIO2 tables have been not demonstrated superiority of either table^4^.

**Table 1:** PEEP/FIO2 tables.

Low PEEP/FIO2 table		High PEEP/FIO2 table	
FIO2 (%)	PEEP (cmH2O)	FIO2 (%)	PEEP (cmH2O)
25–30	5	25–30	5–14
35–40	5–8	35–40	14–16
45–50	8–10	45–50	16–20
55–60	10	55–60	20
65–70	10–14	65–70	20
75–80	14	75–80	20–22
85–90	14–18	85–90	22
95–100	18–24	95–100	22–24

*FIO2* Inspired oxygen fraction, *PEEP* Positive end-expiratory pressure.

Although clinical equipoise currently exists for setting PEEP, some technique for PEEP titration is still needed. The most convenient and user-friendly method remains following the low PEEP/FIO2 table, which was used in the landmark ARMA trial of low versus high tidal volume ventilation^18^. In contrast to quality improvement efforts directed at studying and improving adherence to LTVV, there has been little effort directed at adherence to the low PEEP/FIO2 table. We hypothesize that among patients on LTVV, further adherence to the low PEEP/FIO2 table would be associated with better survival compared to nonadherence. We therefore aimed to study the patient characteristics and clinical outcomes of adherence to the low PEEP/FIO2 table.

## Methods

### Participants and setting

We conducted a retrospective cohort study of patients with ARDS admitted directly from the Emergency Department to our 20-bed Medical ICU from August 2016 to July 2017. We included patients over the age of 21 years old, who were intubated in the Emergency Department prior to ICU transfer, and who had LTVV i.e. tidal volume < 7 ml/kg ideal body weight^2^. Patients were excluded if they were transferred from other locations, as any potential delays in ICU admission might adversely influence patient survival^19^. Patients who were intubated in the ICU after admission were also excluded, as it is uncertain if intubation was delayed^20,21^. Our Ethics Review Board (National Healthcare Group Domain-Specific Review Board) approved the study (National Healthcare Group Domain-Specific Review Board approval number 2018/00223). As the study is a retrospective observational one, the need for patient consent was waived. All procedures and analyses were performed in accordance with relevant guidelines and regulations.

### General clinical care

Patients were initiated on flow-triggered, descending-ramp, volume assist-control as the default mode, using either the Puritan-Bennett 840 or Puritan-Bennett 980 ventilator (Medtronic, MN). Low tidal volumes were used, targeting plateau pressures of less than 30 cmH2O^2^. Recruitment manoeuvers, inhaled nitric oxide, neuromuscular paralysis and prone positioning were seldom used (due to inadequate staffing and protocols). Analgesia was titrated to achieve a Critical‐Care Pain Observation Tool score of 0–2 and sedation was titrated to achieve a Richmond Agitation-Sedation Scale score − 2 to 0. Daily assessment for awakening and spontaneous breathing trials were done. Noradrenaline was the preferred vasopressor, targeting a mean arterial pressure of at least 65 mmHg^22,23^. Sepsis was treated with early, broad-spectrum antibiotics and source control.

### Data collection and definitions

Clinical parameters and arterial blood gas measurements were obtained at the time of ICU admission and at 12 h after ICU admission. Patient outcomes were determined till hospital death or discharge. To determine adherence to the low PEEP/FIO2 table (Table 1), PEEP and FIO2 12 h after ICU admission were used^24^, rather than at the time of ICU admission, to reflect ventilator adjustments by ICU clinicians after initial stabilization. To determine the recommended PEEP, FIO2 values were rounded up to the nearest 10%. For instance, an FIO2 of 55% was taken as 60% on the PEEP/FIO2 table. Applied PEEP at 12 h after ICU admission within the ranges recommended by the low PEEP/FIO2 table was considered adherent, while PEEP above or below the recommended ranges was considered non-adherent.

### Statistical analysis

Proportions, means and medians were compared using Fisher exact, Student t, and Wilcoxon rank-sum tests respectively. We examined the association of adherence to the low PEEP/FIO2 table with age, gender, body-mass index, Acute Physiology and Chronic Health Evaluation (APACHE) II score, primary diagnosis, comorbid conditions, initial arterial oxygen partial pressure to inspired oxygen fraction (P/F) ratio, initial systolic blood pressure, use of vasopressors within the first 24 h of ICU admission, ICU/hospital mortality, ICU/hospital length-of-stay and ventilator-free days through day 28^25^. Logistic regression was used to analyze hospital mortality as an outcome with adherence to the low PEEP/FIO2 as the key independent variable, adjusted for age, APACHE II score, initial P/F ratio and initial systolic blood pressure (adjustment was determined a priori to account for key baseline prognostic factors). For logistic regression analysis, PEEP adherence was additionally coded as a 3-level indicator variable (PEEP lower than recommended; PEEP as recommended; PEEP higher than recommended), with PEEP as recommended being the reference level. Logistic regression was done for all patients with ARDS (P/F ratio 300 mmHg and lower) and repeated for patients with moderate-to-severe ARDS only (P/F ratio 200 mmHg and lower)^1^. Statistical significance was taken as P < 0.05.

This work was performed at the National University Hospital, Singapore.

### Ethics declaration

Our Ethics Review Board (National Healthcare Group Domain-Specific Review Board) approved the study (National Healthcare Group Domain-Specific Review Board approval number 2018/00223). As the study is a retrospective observational one, the need for patient consent was waived.

## Results

**Figure 1:**
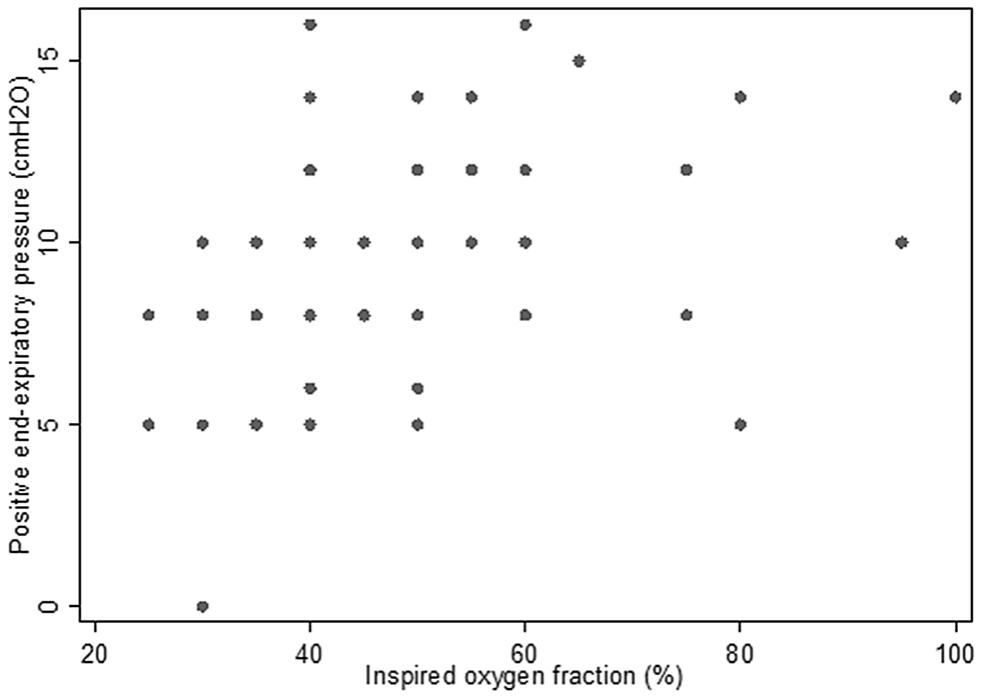
Distribution of PEEP and FIO2 among patients with ARDS.

138 patients with ARDS were analysed: mean age 64.0 ± 26.9 years, 31.2% female, mean APACHE II score 27.9 ± 9.9, mean P/F ratio 145 ± 66 mmHg (Table 2). Distribution of PEEP and FIO2 at 12 h after ICU admission is shown in Fig. 1. Overall adherence to the low PEEP/FIO2 table was 75.4% (Table 3). Of 34 patients in the non-adherent group, 26 (76.5%) had PEEP higher than recommended and these patients also fell into the high PEEP/FIO2 table. Between patients who demonstrated nonadherence to the low PEEP/FIO2 table, compared to those who demonstrated adherence, there were no significant differences found for age, gender, APACHE II score, primary diagnosis, comorbid conditions, initial parameters (P/F ratio, tidal volume corrected for ideal body weight, systolic blood pressure) (Table 2). Compared to patients who received PEEP as recommended by the low PEEP/FIO2 table, a greater proportion of patients who did not receive PEEP as recommended required vasopressors in the first 24 h of ICU admission (40.4% versus 61.8%, P = 0.046). In aggregate, patients who demonstrated nonadherence had 3 fewer ventilator-free days within the first 28 days from ICU admission, spent 3.5 more days in ICU, and had higher ICU (41.2% versus 17.3%) and hospital (52.9% versus 22.1%) mortality. The increased hospital mortality for patients who demonstrated nonadherence persisted after adjustment for age, APACHE II score, initial P/F ratio and initial systolic blood pressure (odds ratio 4.10, 95% confidence interval 1.68–9.99, P = 0.002).

**Table 2:** Patient characteristics and outcomes, by adherence to low PEEP/FIO2 table.

Patient characteristics and outcomes	All patients (N = 138)	Patients with PEEP as recommended by PEEP/FIO2 table (N = 104)	Patients with PEEP not as recommended by PEEP/FIO2 table (N = 34)	P-value
Mean age (years) (SD)	64.0 ± 26.9	64.4 ± 30.0	62.8 ± 13.5	0.764
Female (%)	43 (31.2)	32 (30.8)	11 (32.4)	1.000
Body-mass index (kg/m^2^)	25.5 ± 7.3	25.1 ± 7.3	26.6 ± 7.3	0.316
Mean APACHE II (SD)	27.9 ± 9.9	27.5 ± 10.3	28.9 ± 8.4	0.462
**Primary diagnosis (%)**				0.202
Pneumonia	49 (35.5)	32 (30.8)	17 (50.0)	
Non-pneumonia sepsis	41 (29.7)	34 (32.7)	7 (20.6)	
COPD	1 (0.7)	1 (1.0)	0 (0.0)	
Asthma	2 (1.5)	1 (1.0)	1 (2.9)	
Stroke	7 (5.1)	8 (6.8)	0 (0.0)	
Other^a^	38 (27.5)	29 (27.9)	9 (26.5)	
**Comorbid conditions (%)**				
Diabetes mellitus	78 (56.5)	61 (58.7)	17 (50.0)	0.428
Hypertension	55 (39.9)	41 (39.4)	14 (41.2)	1.000
Ischemic heart disease	36 (26.1)	29 (27.9)	7 (20.6)	0.502
Congestive heart failure	5 (3.6)	5 (4.8)	0 (0.0)	0.333
Asthma	8 (5.8)	5 (4.8)	3 (8.8)	0.407
COPD	8 (5.8)	6 (5.8)	2 (5.9)	1.000
Chronic kidney disease	33 (23.9)	27 (26.0)	6 (17.7)	0.365
Chronic liver disease	24 (17.4)	19 (18.3)	5 (14.7)	0.796
Stroke	3 (2.2)	3 (2.9)	0 (0.0)	1.000
Cancer	16 (11.6)	14 (13.5)	2 (5.9)	0.357
**Initial parameters**				
Mean P/F ratio (mmHg) (SD)	145 ± 66	149 ± 67	134 ± 63	0.274
Mean TV/IBW (ml/kg) (SD)	6.2 ± 0.7	6.3 ± 0.6	6.1 ± 1.1	0.141
Mean Pplat (cmH2O) (SD)	19.5 ± 6.4	19.3 ± 5.7	20.7 ± 9.9	0.508
Mean SBP (mmHg) (SD)	125 ± 31	127 ± 31	120 ± 30	0.244
Use of vasopressors within 1st 24 h of ICU admission (%)	63 (45.7)	42 (40.4)	21 (61.8)	0.046
Median ventilator-free days within first 28 days (IQR)	24 (22–25)	25 (23–25.5)	22 (19–25)	0.002
**Median LOS (days) (IQR)**				
In ICU	6 (4–9)	5 (4–8)	8.5 (5–14)	0.011
In hospital	13 (8–35)	13 (8–39.5)	12 (9–26)	0.980
**Mortality (%)**				
In ICU	32 (23.2)	18 (17.3)	14 (41.2)	0.009
In hospital	41 (29.7)	23 (22.1)	18 (52.9)	0.001
**Odds ratio for hospital mortality (95% CI)**				
Unadjusted	NA	Reference	3.96 (1.75–8.97)	0.001
Adjusted^b^	NA	Reference	4.10 (1.68–9.99)	0.002

*APACHE* Acute Physiology and Chronic Health Evaluation, *CI* Confidence interval, *COPD* Chronic obstructive pulmonary disease, *FIO2* Inspired oxygen fraction, *IBW* Ideal body weight, *ICU* Intensive care unit, *IQR* Interquartile range, *NA* Not applicable, *PEEP* Positive end-expiratory pressure, *Pplat* Plateau pressure, *P/F ratio* Ratio of arterial oxygen partial pressure to inspired oxygen fraction, *SD* Standard deviation, *TV* Tidal volume.

^a^Includes myocardial infarction, bleeding gastrointestinal tract, status epilepticus, drug overdose, pulmonary embolism, diabetic ketoacidosis.

^b^Adjusted for age, APACHE II score, initial P/F ratio, initial systolic blood pressure.

**Table 3:** Adherence to low PEEP/FIO2 table.

FIO2 (%)	Number of patients with PEEP lower than recommended (%)	Number of patients with PEEP as recommended by PEEP/FIO2 table (%)	Number of patients with PEEP higher than recommended (%)	Total number of patients (%)
25–30	1 (2.5)	33 (82.5)	6 (15.0)	40
35–40	0 (0.0)	55 (79.7)	14 (20.3)	69
45–50	2 (12.5)	13 (81.3)	1 (6.3)	16
55–60	2 (25.0)	2 (25.0)	4 (50.0)	8
65–70	0 (0.0)	0 (0.0)	1 (100.0)	1
75–80	2 (67.7)	1 (33.3)	0 (0.0)	3
85–90	No patients	No patients	No patients	No patients
95–100	1 (100.0)	0 (0.0)	0 (0.0)	1
All FIO2	8 (5.8)	104 (75.4)	26 (18.8)	138

*FIO2* Inspired oxygen fraction, *PEEP* Positive end-expiratory pressure.

When PEEP adherence was coded as a 3-level indicator variable (PEEP lower than recommended; PEEP as recommended; PEEP higher than recommended), only PEEP higher than recommended was associated with increased hospital mortality in the whole cohort (adjusted odds ratio 4.51, 95% confidence interval 1.68–12.2, P = 0.003; Table 4) and in patients with moderate-to-severe ARDS (adjusted odds ratio 4.27, 95% confidence interval 1.42–12.8, P = 0.010; Table 5).

**Table 4:** Logistic regression for hospital mortality, by adherence to low PEEP/FIO2 table.

Independent variable	Odds ratio	95% CI	P-value
**Adherence to PEEP/FIO2 table**			
PEEP lower than recommended	2.99	0.59–15.2	0.187
PEEP as recommended	Reference	Reference	Reference
PEEP higher than recommended	4.51	1.68–12.2	0.003
Age (years)	1.01	0.99–1.04	0.295
APACHE II score	1.08	1.03–1.13	0.003
Initial P/F ratio (mmHg)	1.00	0.99–1.01	0.882
Initial systolic blood pressure (mmHg)	0.98	0.97–0.99	0.024

*APACHE* Acute Physiology and Chronic Health Evaluation, *FIO2* Inspired oxygen fraction, *PEEP* Positive end-expiratory pressure, *P/F ratio* Ratio of arterial oxygen partial pressure to inspired oxygen fraction.

**Table 5:** Logistic regression for hospital mortality, by adherence to low PEEP/FIO2 table, for moderate-to-severe ARDS.

Independent variable	Odds ratio	95% CI	P-value
**Adherence to PEEP/FIO2 table**			
PEEP lower than recommended	4.35	0.50–38.0	0.183
PEEP as recommended	Reference	Reference	Reference
PEEP higher than recommended	4.27	1.42–12.8	0.010
Age (years)	1.03	0.99–1.06	0.236
APACHE II score	1.08	1.02–1.14	0.008
Initial P/F ratio (mmHg)	1.00	0.98–1.01	0.478
Initial systolic blood pressure (mmHg)	0.98	0.97–1.00	0.085

Total number of patients with moderate-to-severe ARDS (initial P/F ratio ≤ 200 mmHg) = 105.

*APACHE* Acute Physiology and Chronic Health Evaluation, *FIO2* Inspired oxygen fraction, *PEEP* Positive end-expiratory pressure, *P/F ratio* Ratio of arterial oxygen partial pressure to inspired oxygen fraction.

## Discussion

The main finding of our study is that among patients on LTVV, nonadherence to the low PEEP/FIO2 table was associated with increased mortality compared to adherence, adjusted for baseline critical illness severity, oxygenation and blood pressure (odds ratio 4.10, 95% confidence interval 1.68–9.99, P = 0.002). In particular, applied PEEP higher than that recommended by the low PEEP/FIO2 table was associated with increased mortality (adjusted odds ratio 4.51, 95% confidence interval 1.68–12.2, P = 0.003). Patient characteristics at baseline were not associated with adherence to the low PEEP/FIO2 table.

Possible explanations for the association of mortality with PEEP/FIO2 table non-adherence could be the deleterious pulmonary and cardiovascular effects of high PEEP. High PEEP may cause overdistension of alveoli^7^, possibly leading to biotrauma and barotrauma, though we did not observe the latter in our patients. In addition, high PEEP can increase intrathoracic pressure, impede venous return, reduce cardiac output, and cause systemic hypotension^8^. In our patients, these might have occurred. Patients who were not adherent to the low PEEP/FIO2 table generally received higher PEEP than recommended and a greater proportion required vasopressors in the first 24 h of ICU admission. Given that association does not mean causation, one needs to consider whether reverse causation was possible i.e. a patient who has higher mortality drove the use of higher PEEP. We feel that this possibility is slim since none of the baseline prognostic factors were associated with nonadherence to the low PEEP/FIO2 table.

Other possible explanations for the mortality difference between the adherent and non-adherent groups were considered. Firstly, based on peripheral oxygen saturation/FIO2 (S/F ratio) changes, we classified patients with decreased S/F ratio over the first 12 h as deteriorating, and patients with stable or improved S/F ratio as non-deteriorating. The proportion of deteriorating patients was similar between the non-adherent and the adherent groups (6/34 [17.7%] versus 15/104 [14.4%], P = 0.783). As such, the higher mortality in the non-adherent group could not be explained by a higher proportion of deteriorating patients. Secondly, among 85 patients with plateau pressure measured at 12 h, between the adherent and non-adherent groups, the mean driving pressure was not significantly different (13.3 versus 11.2 cmH2O, P = 0.307) and could not explain the difference in mortality. Thirdly, although the absolute difference was large, pneumonia as the etiology of ARDS was not statistically different between the adherent and non-adherent groups (P = 0.062). The increased hospital mortality for patients who demonstrated nonadherence persisted after adjustment for age, APACHE II score, initial P/F ratio, initial systolic blood pressure *and* pneumonia as the ARDS etiology (odds ratio 5.90, 95% confidence interval 2.14–16.2, P = 0.001).

In our study, applying PEEP lower than that recommended by the low PEEP/FIO2 table was not significantly associated with mortality for a few reasons. Firstly, all patients except one had PEEP of at least 5 cmH2O and severe decruitment in most patients would be unlikely. Secondly, it may be possible that low PEEP may be sometimes beneficial, for instance to avoid excessive right ventricular afterload and to reduce the risk of acute right ventricular failure. Thirdly, only 5.8% of patients had PEEP lower than recommended by the low PEEP/FIO2 table, compared to triple the proportion of patients (18.8%) with PEEP higher than recommended by the low PEEP/FIO2 table, which meant that the power to detect a significant association for the former group of patients would be limited. Just like for pediatric ARDS^26^, real harm from setting PEEP lower than that recommended by the low PEEP/FIO2 table could also exist in adult ARDS.

The lack of association between baseline patient characteristics and adherence to low PEEP/FIO2 table is not surprising, given clinical equipoise over PEEP optimization. In our ICU, while there has been broad consensus over the need to limit tidal volume and driving pressure, and guidelines to keep peripheral oxygen saturation between 90–96%, there has been no firm recommendation about PEEP titration. Esophageal balloon catheters and electrical impedance tomography are unavailable, leaving only three options for clinicians: pressure–volume curve based titration, lung ultrasound and the low PEEP/FIO2 table. Anecdotally, the low PEEP/FIO2 table was the most convenient tool and as expected, most people followed it.

Our observational study differs from prior randomized trials of high versus low PEEP in ARDS. Our study does not suggest that low PEEP is better than high PEEP, nor does it suggest that personalization of PEEP using other methods cannot not used. Rather, our results suggest that if one sets PEEP using the low PEEP/FIO2 table, then adherence is associated with reduced hospital mortality, supporting the use of the low PEEP/FIO2 table to guide PEEP setting for mechanically ventilated patients with ARDS. In addition, our results highlight the need to study adherence to the low PEEP/FIO2 table as a quality assurance metric.

If nonadherence to the PEEP/FIO2 table is found, several methods could be used to improve adherence. Manual methods include implementation of a management protocol for clinicians^27,28^ or an order set driven by respiratory therapists^24^. Semi-automated methods include using a computer-assisted oxygen advisor^29^ or a closed-loop system^30^. As can be surmised, prospective studies demonstrating the clinical impact of improving adherence to the PEEP/FIO2 are needed.

While our study may be one of the first exploring the association of PEEP/FIO2 table adherence with clinical outcomes, we acknowledge some limitations. Firstly, we performed our study in a single center, and in a tertiary-level medical ICU experienced with mechanical ventilation for ARDS. This may limit generalizability. However, such a setting was crucial to help us investigate variations of clinical outcomes with variations of PEEP setting, while having taken care of important prognostic factors like low tidal volume and oxygenation management. Secondly, we only studied the PEEP and FIO2 adherence at 12 h after ICU admission. Nonetheless, adherence at this time point was associated with adherence at a later time point. In our cohort, among the 104 patients adherent to the low PEEP/FIO2 table at 12 h after ICU admission, 82 (78.9%) remained adherent and 22 (21.2%) were non-adherent at 24 h (McNemar’s P = 0.638). Conversely, among the 34 patients non-adherent to the low PEEP/FIO2 table at 12 h after ICU admission, a higher proportion of patients (18 patients, or 52.9%) switched adherence status (i.e. became adherent at 24 h). Although this higher proportion would predispose any observed association of adherence and mortality towards the null, our analysis still turned in a significant association. Thirdly, given the distribution of PEEP and FIO2 in our cohort, which reflected general use of the low PEEP/FIO2 table among our ICU clinicians, we could not investigate adherence to the high PEEP/FIO2 table. Fourthly, our results may not apply to patients who are severely obese^31^ or who have raised intra-abdominal pressure^32^. Fifthly, we did not have reliable recordings of peak inspiratory pressure and inspiratory flow and could not compute the mechanical power for further investigation.

## Conclusions

In conclusion, adherence to the low PEEP/FIO2 table was associated with better survival compared to nonadherence, among mechanically ventilated patients with ARDS who received LTVV. This suggests that for PEEP setting, in lieu of more sophisticated methods, guidance using the low PEEP/FIO2 table remains clinically meaningful^17^. Similar studies should be performed in other ICUs to confirm our results. Upon confirmation, quality assurance for mechanical ventilation among patients with ARDS should include steps to monitor and improve adherence to the low PEEP/FIO2 table.

## Data Availability

No consent to share data could be obtained.
